# Comparison of Intramedullary Magnetic Nail, Monolateral External Distractor, and Spatial External Fixator in Femur Lengthening in Adolescents with Congenital Diseases

**DOI:** 10.3390/jcm10245957

**Published:** 2021-12-18

**Authors:** Szymon Pietrzak, Dariusz Grzelecki, Tomasz Parol, Jarosław Czubak

**Affiliations:** 1 Department of Orthopedics, Pediatric Orthopedics and Traumatology, Centre of Postgraduate Medical Education, Professor Adam Gruca Orthopedic and Trauma Teaching Hospital, Konarskiego 13, 05-400 Otwock, Poland; tparol@gmail.com (T.P.); czubakjarek@gmail.com (J.C.); 2 Department of Orthopedics and Rheumoorthopedics, Centre of Postgraduate Medical Education, Professor Adam Gruca Orthopedic and Trauma Teaching Hospital, Konarskiego 13, 05-400 Otwock, Poland; dariuszgrzelecki@gmail.com

**Keywords:** lower limb discrepancy, limb lengthening, Taylor Spatial Frame, intramedullary growing nail, monolateral external distractor

## Abstract

The aim of this study is to evaluate the course of the treatment and clinical and functional outcomes of femur lengthening in adolescents with congenital disorders by the application of different surgical methods. This retrospective study comprised 35 patients (39 procedures). A total of 11 patients underwent femur lengthening with the use of the intramedullary magnetic nail (IMN) Precise 2 (NuVasive, San Diego, CA, USA), 7 patients (11 procedures) with the use of the monolateral external distractor Modular Rail System (MRS) (Smith and Nephew, Memphis, TN, USA), and 17 with the use of the computer-assisted external fixator Taylor Spatial Frame (TSF) (Smith and Nephew, Memphis, TN, USA). The inclusion criteria were as follows: (1) congenital femoral length deficiency without any axial deformities and (2), independently of the finally applied treatment, the technical possibility of use of each of the analyzed methods. The distraction index did not differ significantly between the groups (*p* = 0.89). The median lengthening index was the lowest in the IMN group (24.3 d/cm; IQR 21.8–33.1) and statistically different in comparison to the MRS (44.2 d/cm; IQR 42–50.9; *p* < 0.001) and the TSF groups (48.4 d/cm; IQR 38.6–63.5; *p* < 0.001). Similarly, the consolidation index in the IMN group (12.9 d/cm; IQR 10.7–21.3) was statistically lower than that in the MRS (32.9 d/cm; IQR 30.2–37.6; *p* < 0.001) and the TSF (36.9 d/cm; IQR 26.6–51.5; *p* < 0.001) groups. This study indicates that IMN is a more valuable method of treatment for femoral length discrepancy without axial deformity than MRS and TSF in complication rate and indexes of lengthening and consolidation.

## 1. Introduction

Lower limb discrepancy (LLD), which may cause static and dynamic disorders of the whole body, is a serious functional problem in children and adolescents. It is estimated that a lower limb length inequality of up to 1.5 cm affects one-third of the population and that greater than 2 cm occurs in 7% of healthy children aged from 8 to 12 years old [1,2]. Based on observational studies, it was assumed that a 2 cm value of deformity is maximal for pelvic and postural balance compensation [3,4]. However, some authors emphasize that even this “acceptable” threshold should be adopted with caution and analyzed considering individual anatomical proportions and proprioceptive capacity [5]. Nevertheless, disproportion over the mentioned limits in children demands the particular assessment of the influence on the musculoskeletal system, the prognosis of the rise with age, and the choice of the proper method of treatment [6].

Currently, in patients with a significant congenital lower limb length deficiency, the commonly used treatment strategies include surgical bone lengthening [5,6,7]. For this purpose, external fixators (monolateral and spatial) and intramedullary growing nails are used [8,9,10,11,12]. The choice of the best method depends on several coexisting factors, such as age, additional axial and rotational bone deformities, and technical issues of the application of the specific implant (e.g., the presence of active physis in the entry point and a sufficient diameter of the bone marrow cavity allowing for the implantation of the intramedullary nail) [9,10,13].

Adverse effects are an additional aspect that should be considered before the application of the treatment strategy. In terms of surgical limb lengthening, the classification of these incidences based on problems, obstacles, and serious complications remains justified [14]. Besides the long period of time in the frame, the most frequent adverse effects of external fixators include pin-site infections; implant loosening and frame destabilization; and problems with regeneration, such as premature consolidation, non-union or malunion, limited range of motion (ROM), and joint stiffness [15]. Using intramedullary growing nails avoids some of these issues, but other serious complications, such as deep infections and the breakage of locking screws and the nail itself, may appear more frequently [16,17,18,19]. A comparison of the clinical and functional outcomes of different methods of limb lengthening is essential, especially in patients for whom more than one strategy can be used to achieve limb equalization. However, there is still a lack of comprehensive studies focusing on this issue. 

The aim of this retrospective study is to evaluate the course of the treatment and clinical and functional results of femur lengthening in adolescents without axial and rotational deformity with congenital disorders by the application of different methods: (1) intramedullary growing nails, (2) monolateral external distractors, and (3) computer-assisted circular external fixators. The intention of this study is to compare these three methods in patients who would be potential candidates for each of the analyzed implants on the basis of preoperative clinical examination and radiological assessment.

## 2. Materials and Methods

**Figure 1:**
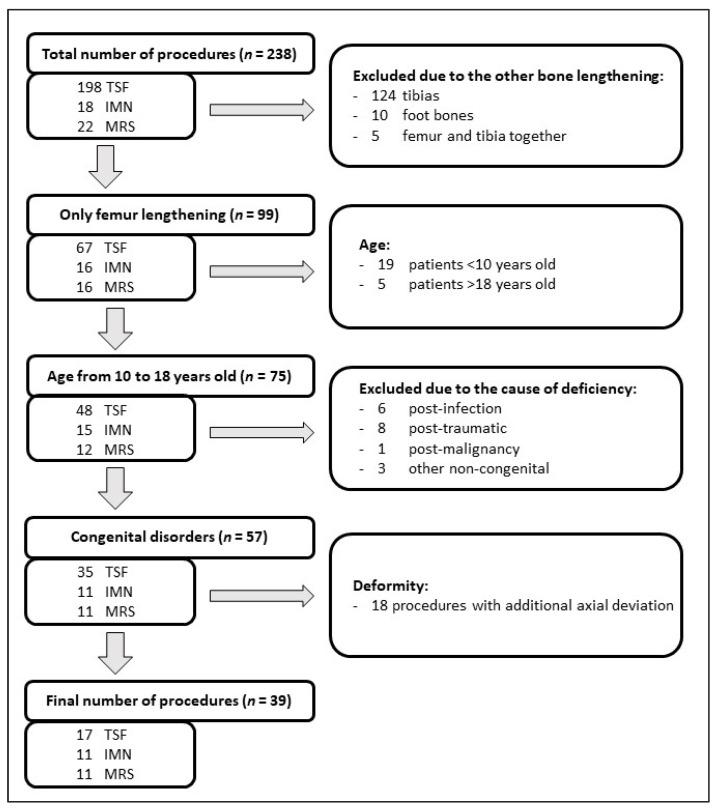
Flow diagram of inclusion and exclusion criteria. TSF—Taylor Spatial Frame; IMN—intramedullary magnetic nail; MRS—Modular Rail System.

A total of 186 patients (238 procedures) operated on in the single children’s orthopedic department from April 2015 to December 2020, who underwent bone lengthening treatment, were retrospectively analyzed in terms of inclusion and exclusion criteria. The following criteria were used for inclusion in the study: (1) femoral length deficiency caused by congenital diseases without any axial deformities and (2), independently of the finally applied treatment, the technical possibility of use of each of the analyzed methods. Exclusion criteria were established as <10 and >18 years old and no technical possibility to apply any of the tested methods in the retrospective assessment, e.g., a too-narrow medullary canal for nail implantation, axial deformity of the femur bone before the treatment, acquired femoral length deficiency due to malignancy, infection, and/or fracture. Finally, this study included 35 patients (39 procedures), with 11 procedures using intramedullary magnetic nails (IMNs) (Precise 2 (NuVasive, San Diego, CA, USA)), 11 procedures (7 patients) using monolateral external distractors (Modular Rail System (MRS) (Smith and Nephew, Memphis, TN, USA)), and 17 procedures using external fixators (Taylor Spatial Frame (TSF) (Smith and Nephew, Memphis, TN, USA)). A flow diagram of the inclusion and exclusion criteria is presented in Figure 1. 

All procedures were performed by two experienced surgeons from the children’s orthopedic department, with the use of the same surgical technique and rehabilitation protocol for each method. Before the surgery, digital long-standing radiographs were taken. To determine the aim of lengthening, the LLD was calculated in millimeters and compared to the contralateral limb or the program plan if both limbs and different segments required lengthening. The choice of treatment method was made in relation to indications and the availability of the implants in the department of the first author in the analyzed period. TSFs were available for the entire duration of the study and were used between April 2015 and December 2020. MRS’ were used in 2019 (8 procedures) and in 2017 (3 procedures). IMN, as a newly introduced method, was applied from January to November 2020. All patients were mobilized the day after surgery and encouraged to ambulate with walking aids. Physical therapy started at the same time in order to maintain a good range of motion in the knee. 

The lengthening started on the 7th day after surgery, with the standard value of 1 mm per day (1 mm × 1 mm for the TSF group, 4 mm × 0.25 mm for the MRS group, and 3 mm × 0.33 mm for the IMN group). The tempo of distraction was adjusted depending on the patient’s tolerance, maintaining the appropriate knee joint ROM and radiographic features of regenerate formation. Full weight bearing was allowed in the TSF and MRS groups right after the surgery; full weight bearing for patients treated with IMN was not permitted during the distraction phase, but partial weight bearing was introduced from the beginning of the consolidation phase. Full weight bearing was permitted from the day when 3 of 4 cortices were present on the antero-posterior and lateral radiographs. No casting or bracing was used after the surgery. The patients were examined in an outpatient clinic every two weeks during lengthening (including radiographic assessment) and then once a month during the consolidation phase. The recorded adverse events were assessed as the focal point of treatment evaluation and were divided into problems, obstacles, and complications in accordance with the Paley classification [14]. Three indexes, namely, the distraction index (length of the regenerate in mm divided by the time of distraction in days), the lengthening index (time from the surgery to full weight bearing in days divided by the length of regenerate in cm), and the consolidation index (time from the last day of distraction to the consolidation in days divided by the length of regenerate in cm), were calculated and compared for all groups.

Statistical analysis was performed with the use of STATISTICA 13 Software (Tibco Software Inc., Palo Alto, CA, USA). The Shapiro–Wilk test was performed to determine the normality of the data. Due to the small groups of patients and the lack of normal distribution, the Kruskal–Wallis ANOVA test and Mann–Whitney U test were used to calculate the statistical relationships between the demographical and clinical data. The χ2 test (2 × 3) was carried out to assess the differences in dichotomous variables. The significance level was adopted as *p* < 0.05.

## 3. Results

Significant differences between the groups regarding median age (*p* = 0.65) and weight (*p* = 0.9) were not observed. However, median BMI results differed significantly (*p* = 0.002), with higher median values in the MRS group (26.7 kg/m^2^; IQR 22.9–27.3) than those in the IMN (22.5 kg/m^2^; IQR 19.4–23.6; *p* = 0.022) and in the TSF groups (21.8 kg/m^2^; IQR 19.3–22.7; *p* = 0.001). The longest median operation time was noted in the IMN group (125 min; IQR 113–130) in comparison to the MRS (80 min; IQR 55–89; *p* < 0.001) and TSF (101 min; IQR 80–115; *p* = 0.011) groups. Comparing the MRS and TSF groups, a significantly longer operative time was noted in the TSF group (*p* = 0.03). Insignificant differences in distraction aim and final achieved distraction were observed among the groups (*p* = 0.07 and *p* = 0.08, respectively). The lengthening goal was achieved in 35/39 of included patients (89.7%). In the IMN group, the mean correction index was 98.3% (±3.01); in the MRS group, it was 99.4% (±1.4); and in the TSF group, it was 100%.

Despite this, the distraction index did not differ significantly between the groups (*p* = 0.89). The median lengthening index was the lowest in the IMN group (24.3 d/cm; IQR 21.8–33.1) and statistically different in comparison to the MRS (44.2 d/cm; IQR 42–50.9; *p* < 0.001) and the TSF groups (48.4 d/cm; IQR 38.6–63.5; *p* < 0.001). Similarly, the consolidation index in the IMN group (12.9 d/cm; IQR 10.7–21.3) was statistically lower than that in the MRS (32.9 d/cm; IQR 30.2–37.6; *p* < 0.001) and the TSF (36.9 d/cm; IQR 26.6–51.5; *p* < 0.001) groups. Time from the surgery to hardware removal was not assessed in the IMN group because nail extraction was not obligatory after the regenerate consolidation; thus, allowing full weight bearing was treated as the endpoint of the treatment in this group (Table 1). 

**Figure 2:**
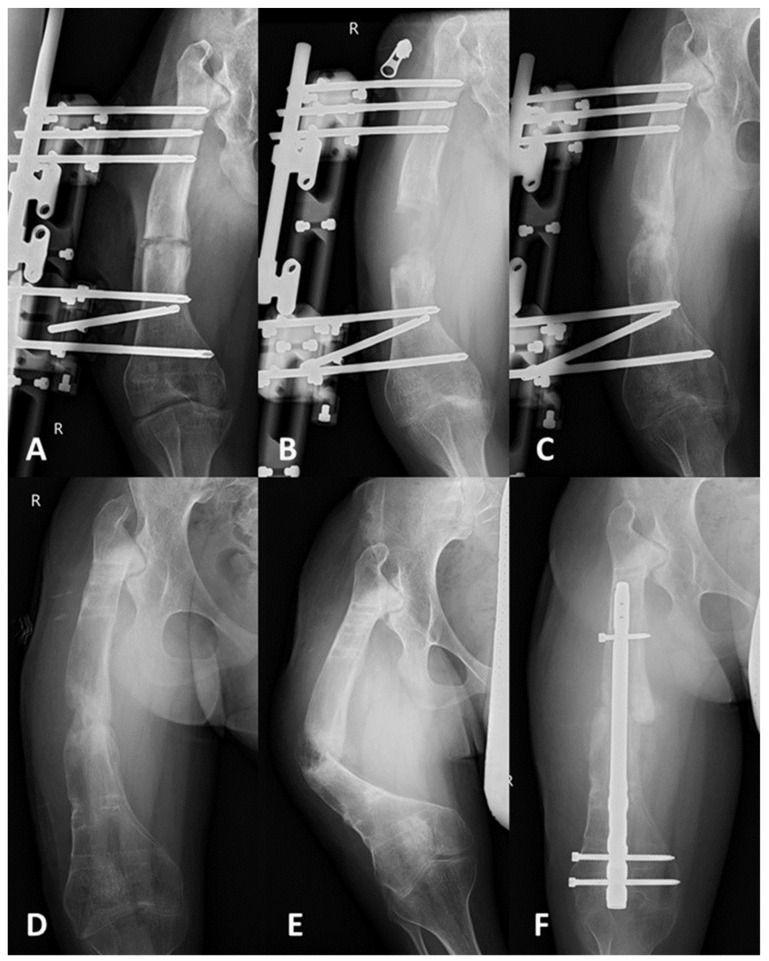
AP radiographs of a 15-year-old patient with CFD and 45 mm aim of lengthening, qualified for femur lengthening with external fixator MRS. (**A**) Postoperative AP radiograph. (**B**) AP radiograph after the end of distraction (before the regenerate consolidation). (**C**) Partial regenerate consolidation. (**D**) Progress in consolidation, radiograph after the frame removal. (**E**) Regenerate fracture was observed 27 days after the frame removal and recommended partial weight bearing in a cast. (**F**) Fracture stabilization with retrograde solid nail—2 mm distraction was subsequently lost to this treatment.

**Figure 3:**
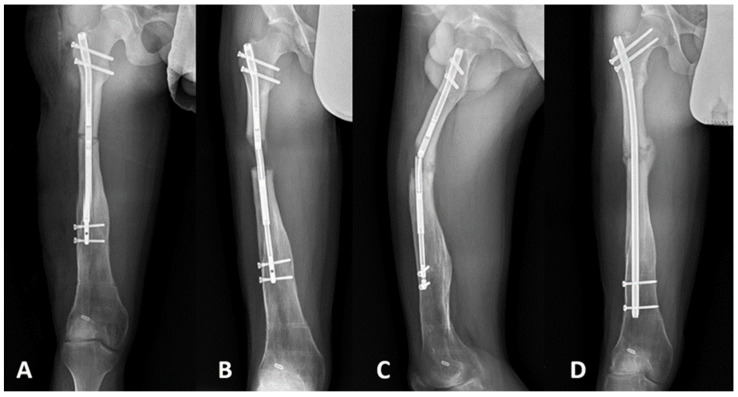
AP radiographs of a 15-year-old patient with CFD and 50 mm LLD qualified for femur lengthening with IMN Precise 2. (**A**) Postoperative AP radiograph. (**B**) AP radiograph after the end of distraction (before the regenerate consolidation). (**C**) Exposed broken IMN and regenerate. The patient did not follow the recommendations and went on a hike in mountains and started full weight bearing before the regenerate consolidation. (**D**) Fracture stabilization with the use of solid femoral nail—5 mm distraction was subsequently lost.

The adverse effects of the therapy were observed and analyzed. The highest total rate of problems was noted in the TSF and MRS groups. At the pin sites of those in these groups, superficial infections were observed in five patients (29%) and two patients (18%), respectively. Moreover, one patient in the TSF group (6%) expressed painful heterotopic intramuscular ossifications in the pin site places. We found obstacles after the application of each method, including delayed consolidation in the IMN group (one patient; 9%), frame destabilization (two patients; 18%) and pre-consolidation (one patient; 9%) in the MRS group, and bone bending (two patients; 12%) in the TSF group. Serious complications were fracture post frame removal (one patient; 9%; Figure 2) and malunion union (one patient; 9%) in the MRS group, two fractures post TSF removal (12%), and hardware failure—broken IMN and regenerate—in one patient (9%; Figure 3) (Table 2).

## 4. Discussion

Limb lengthening in adolescents with congenital etiology remains a clinical challenge for orthopedic surgeons. Previously conducted clinical and experimental studies emphasize that untreated, significant LLD later in adulthood may affect the motor functions of the whole body [4,6]. However, several reports suggest that even mild LLD from 0.5 cm to 1 cm, which should be compensated, may negatively influence gait kinematics and cause its deviations [20]. Gurney et al. showed that an artificial limb length disproportion of 2 cm or more may cause an increase in oxygen consumption and perceived physical exertion and, over 3 cm, may influence other physiological parameters, such as quadriceps activity, heart rate, and minute ventilation [21]. Thus, indications for limb lengthening and/or epiphysiodesis of the contralateral segment should be made individually subsequently to clinical examination, assessment of limb inequality, patient expectations, and precise preoperative planning [22].

The variety of bone lengthening methods creates the need to define the indications of use for each of them. IMNs are strongly technically limited to children and adolescents with a sufficient bone canal diameter and physeal arrest. In a single report, Dahl et al. described off-label, extramedullary motorized nail use for femur lengthening in 11 children below the age of 8 years old [13]. They concluded that lengthening of the femur with this technique may be achieved safely in young children, despite the fact that most of the cases (7/11 cases) presented small varus deformity or bone procurvatum through the regenerate and that 3 out of 11 cases required unplanned reoperation. Our results concerning the lengthening index for each subgroup correspond to those observed in many studies [23,24,25,26]. We observed more favorable values for the consolidation index for the IMN subgroup than for the ex-fix subgroups, which is also reported elsewhere [9,27]. Our study focused on adolescents and indicated excellent performance with a mean ranging from 98.3% to 100% of expected lengthening depending on the applied method. In one patient in the IMN group, 5 mm distraction was lost due to nail and regenerate fractures. However, this patient did not follow the recommendations. He went on a hike in mountains and started full weight bearing before the regenerate consolidation (Figure 3). 

Schiedel et al. assessed the precision of the Precise 2 nail, and, among their cohort, 12 patients were ≤18 years old [17]. The mean lengthening accuracy ratio was 98.3%, which is the same rate as in our study. Our results are in line with other authors who compared IMN and monolateral external fixators for lower limb lengthening [9,28]. Szymczuk et al. emphasized that the intramedullary method represents a significant improvement to monolateral external fixators [9]. In the MRS group, the lengthening goal was achieved in 88% of patients and in the IMN group in 87% of patients. Generally, the “internal” method of lengthening avoids the most common problems—pin tract infections and limited range of motion in adjacent joints reported in patients with external fixation. Statistically significant differences in terms of adverse events (81% in the MRS group vs. 60% in the IMN group; *p* < 0.001), limited ROM at the end of distraction (*p* < 0.0007), and post-consolidation (*p* < 0.0001) were observed. 

Shabtai et al. reported complications after lengthening with a motorized intramedullary nail (Precise^®^) in patients with congenital femoral deficiency (CDF) and fibular hemimelia (FH) [25]. In total, 6 out of 21 patients who underwent femoral lengthening required surgery afterward due to hip flexion contracture, hip subluxation, knee subluxation, and two delayed unions. They did not report any hardware failure. In our study, we faced a smaller number of complications in this subgroup; this may have been because not only CFD cases were included. Kirane et al. also reported less adverse events (1 implant failure and 6 non-implant-related problems in 24 patients), but in their group, a varied etiology of limb shortening was included [29]. Similarly, Black et al., who analyzed the results of femoral lengthening in skeletally mature children with congenital diseases, indicated a decreased “Category-I” complication rate (pin-track infection and mild joint contractures, which require minimal intervention) in the motorized nail group in comparison to the circular frame group (0.4 vs. 1.1 per lengthening session, *p* = 0.02) [10]. The rate of more severe complications was similar in both groups. Horn et al. also observed a larger number of complications in patients treated with circular frames than in those treated with a motorized nail [27]. 

The modification of the intramedullary technique was proposed by Paley et al., who combined a monoliteral external fixator with intramedullary nail splinting and compared it with the Ilizarov method [15]. They did not observe significant differences in clinical outcomes between the study groups (*p* = 0.37) but emphasized its advantages of lengthening over an intramedullary nail in the decrease in the duration of external fixation, protection against refracture, and earlier rehabilitation. Using this combined technique, the other authors confirmed its usefulness in the lengthening of the femur and tibia, although the rate of deep infections remains high, from 2.4% to 15% [30,31,32]. 

In our material, superficial pin-site infections were noted in seven cases of external fixators (25%), while deep infections were not observed. The main advantage of TSF over monolateral external fixators is the possibility to correct axial deformations simultaneously with bone distraction. This computer-assisted method is a valuable tool for bone distraction with the correction of complex deformities. Fracture of the regenerate remains a significant complication of bone lengthening. Four events of fractures occurred: two in the TSF group, one in the MRS group, and one in the IMN group with concomitant nail fracture. Horn et al. indicate that the rates of secondary surgeries and complications in patients with congenital disorders (mostly due to fracture after hardware removal and lack of healing regenerate) are higher than in those with acquired etiology [33]. Thus, in the case of external fixator use, additional prophylactic intramedullary stabilization with titanium nails, Rush rods, or nails is a promising solution to avoid fracture after frame removal [34,35,36,37]. Besides the splinting on the standard intramedullary nails, Popkov et al., in their multicenter study, compared hydroxyapatite-coated nails and titanium elastic nails for that purpose and revealed a decreased external fixation index and good to excellent results [35,36]. Moreover, there was a statistically significant negative correlation between nail diameter and fracture occurrence after frame removal, which is an important factor that has to be accounted for in this treatment strategy [36]. 

We observed a statistically significant higher mean value of BMI in the MRS group than in the other two groups. This may be due to the etiology of shortening in these patients (7 of 11 patients with achondroplasia or hypochondroplasia; in the TSF subgroup, only 1 patient with achondroplasia). Comparing the results of the consolidation index and the distraction index between the two ex-fix subgroups, in our opinion, no impact of higher BMI on lengthening results can be established. 

The aspects being increasingly discussed are quality of life and tolerance of the applied method of LLD treatment. Currently, there is no scale dedicated to the pediatric population, and the PedsQL scale has been adopted in several studies [38,39]. The evident disadvantages of an external fixator are the presence of a frame, which hinders daily activity and exercise with the need for everyday pin-site cleaning, and a second surgical procedure for hardware removal. In the case of IMN, removal is not obligatory. Additionally, some doubts concern the possibility of magnetic resonance imaging (MRI) subsequently to IMN application. Despite this, it has not been tested for compatibility in the MRI environment and did not receive approval from the Federal Drug Agency (FDA); several studies have tested the safety from this aspect. Gomez et al. did not find negative effects, such as heating, elongation, and migration forces, acting upon this implant in 1.5T and 3T fields [40]. Nevertheless, they concluded that 3T protocols should be avoided in patients who are still undergoing lengthening or if lengthening is planned in the future. 

Our study has some limitations, which should be considered before the analysis of the results. Firstly, the small size of the examined groups is evident. We decided to only include patients with congenital etiology of the femur length deficiency. Post-infected and post-traumatic cases and patients with malignancies were excluded due to adverse influence on the femur lengthening process and the potentially higher frequency of complications. Additionally, the set of inclusion and exclusion criteria making the groups homogenous, especially in terms of the availability for the application of all three methods of lengthening, is justified for proper comparison and to avoid bias in the interpretation of the results. The second limitation is the retrospective nature of this study and the lack of randomization. However, the compared implants were only available during a certain period (MRS and IMN). The design of the study with the selection of patients who could be treated with all implemented methods assessed in this research can correspond to the randomization. Lastly, the time of follow-up was different, but this was insignificant. This was caused by the implantation of the hardware at various timelines and implant availability. The shortest observation time was in the IMN group, but there was no need to remove the hardware before full weight bearing, and all patients excluding complicated cases achieved regenerate consolidation.

## 5. Conclusions

In conclusion, our study indicates IMN as the most valuable method of treatment for femoral length discrepancy without axial deformity. The strongest advantages were noted in the lowest rate of adverse effects (especially problems and obstacles) and faster regenerate organization with a return to full weight bearing, but a potentially more invasive procedure of hardware removal. We believe that IMNs and TSFs are currently the best options for simple femur bone lengthening in adolescents with congenital disorders. However, there is a need to confirm our findings in a larger group of patients with the randomization protocol. 

## Figures and Tables

**Table 1:** Demographical and clinical data. Continuous values are presented as medians with interquartile range (IQR).

	IMN (*n* = 11)	MRS (*n* = 11)	TSF (*n* = 17)	*p*-Value
Males/Females	5/6	7/4	9/8	0.69 *
Age (years)	14 (11–15.5)	14 (13–15.5)	13.6 (11–16)	0.65 **
Weight (kg)	56 (53–58.8)	54 (42–62.5)	53 (46.5–63.5)	0.9 **
BMI (kg/m^2^)	22.5 (19.4–23.6)	26.7 (22.9–27.3)	21.8 (19.3–22.7)	0.002 **
Hospital stay (days)	12 (10.5–13.8)	9 (9–11)	10.3 (9–12)	0.19 **
Time of surgery (min)	125 (113–130)	80 (55–89)	101 (80–115)	<0.005 **
**Distraction parameters**				
Distraction aim (mm)	50 (33.5–54.5)	55 (45–55)	42 (30–50)	0.07 **
Achieved distraction (mm)	45 (33.5–53.8)	55 (45–55)	42 (30–50)	0.08 **
Distraction time (days)	45 (33–53)	55 (53–59)	42 (29–51)	0.03 **
Distraction index (mm/d)	0.85 (0.81–0.86)	0.85 (0.73–0.89)	0.85 (0.83–0.88)	0.89 **
Lengthening index (days/cm)	24.3 (21.8–33.1)	44.2 (42–50.9)	48.4 (38.6–63.5)	<0.001 **
Consolidation index (days/cm)	12.9 (10.7–21.3)	32.9 (30.2–37.6)	36.9 (26.6–51.5)	<0.001 **
Time to device removal (days)	N/A	179 (172.5–195.8)	150 (128–165)	0.002 ***
Time to full weight bearing (days)	106 (90–118)	227 (202–232)	174 (152–214.5)	0.001 **
Follow-up (months)	10 (9.5–13.5)	17 (10.5–25)	15 (9–21.5)	0.2 **
**Diagnosis**				
CFD	7	3	10	
Achondroplasia	-	6	-	
Hypochondroplasia	-	1	1	
Fibular hemimelia	-	1	3	
Congenital dislocation of the hip	2	-	3	
Beckwith–Wiedemann syndrome	2	-	-	

* χ2 test; ** Kruskal—Wallis ANOVA test; *** Mann—Whitney U test.

**Table 2:** Problems, obstacles, and complications among the study groups.

	IMN (*n* = 11)	MRS (*n* = 11)	TSF (*n* = 17)
**Problems**	None	**2 (18%)**—pinsite/superficial infection	**5 (29%)**—pin site/superficial infection **1 (6%)**—heterotopic intramuscular ossifications in pin places
**Obstacles**	**1 (9%)**—delayed consolidation	**2 (18%)**—frame Destabilization **1(9%)**—pre-consolidation	**2 (12%)**—bone bending
**Complications**	**1 (9%)**—hardware failure (broken nail and regenerate fracture) (Figure 2)	**1 (9%)**—malunion **1 (9%)**—fracture post removal (Figure 3)	**2 (12%)**—fracture post removal

## Data Availability

The datasets generated and analyzed in the current study are available from the corresponding author on reasonable request.
